# Event and Comment

**Published:** 1917-11

**Authors:** Homer C. Brown

**Affiliations:** Chairman Legislative Committee, N. D. A.


### DENTAL REGISTER

Vol. LXXI NOVEMBER, 1917 No. 11

### EVENT AND COMMENT

OUR NEW DENTAL CORPS.—By concurrence of the House in Senate amendments to House Bill 4897 in the closing hours of Congress, October 6, a Dental Corps has been created in the Army, corresponding to the Medical Corps, the membership of which shall have the rank, pay, promotion and allowance of officers of and shall be of the corresponding grade of the Medical Corps. Chairman Dent of the House Military Committee explained that the House bill relating to members of the Dental Service of the Army corresponded to the section of the national defense law which waived the former requirement of the law of five years' previous service in the Army as lieutenant before a member could be promoted to the next higher rank, and so on for the higher grades. The Senate practically adopted the House bill with the material amendment that a Dental Corps be created in the Army. Heretofore, the highest rank a member of the dental service could attain was that of major, and it would require twenty-four years of service before he could obtain that rank. At present the highest officers in the Army dental service have the rank of captain.

We are indebted to Dr. H. C. Brown, of Columbus, for a copy of the legislation for which he has worked indefatigably for several months, and the profession is under great obligations to him and the other members of the committee for their faithful and untiring efforts to get the legislation through Congress. We print that portion of the bill below which gives a brief statement of the new rank of the dentist in the Army.

### 65TH CONGRESS, 1ST SESSION, H. R. 4897. IN THE SENATE OF THE UNITED STATES, AMENDMENT PROPOSED BY MR. POMERENE.

After the word "War" (which is the closing word in H. R. 4897) insert the following:

"Hereafter the Dental Corps of the Army shall consist of commissioned officers of the same grades and proportionally distributed among such grades as are now or may be hereafter provided by law for the Medical Corps, who shall have the rank, pay, promotion, and allowances of officers of corresponding grades in the Medical Corps, including the right to retirement as in the case of other officers, and there shall be one dental officer for every thousand of the total strength of the Regular Army authorized from time to time by law: Provided further, That dental examining and review boards shall consist of one officer of the Medical Corps and two officers of the Dental Corps: And provided further, That immediately following the approval of this act all dental surgeons then in active service shall be recommissioned in the Dental Corps in the grades herein authorized in the order of their seniority and without loss of pay or allowances or of relative rank in the Army: Provided further, That no dental surgeon shall be recommissioned who has not been confirmed by the Senate. All regulations concerning the enlistment of medical students in the Enlisted Reserve Corps and their continuance in their college course while subject to call to active service shall apply similarly to dental students."

The above is an exact copy of our new legislation. This last provision, as well as the one immediately preceding it, relating to the recommissioning of dental surgeons, was added while the original amendment was before the Senate for consideration. All preceding these last two provisions, is what we have termed the Lodge Amendment as modified, and was introduced by Senator Pomerene. In explanation will say that unfortunately Senator Lodge left Washington about two weeks before Congress adjourned. Our friends advised that considerable opposition had developed and suggested that our amendment be made as short as possible. Further, we desired to add a very few words in a couple of places, in order to strengthen our legislation, endeavoring to make it impossible to leave things for too much interpretation. In shortening the Lodge Amendment, we eliminated three lines relating to the examination for officers of Dental Corps, because it is already provided by law. The last provision of the Lodge Amendment was entirely eliminated. This related to promotion of first lieutenants of the Medical Department, as, under existing conditions, it was more or less superfluous, and might have weakened our cause.

HOMER C. BROWN,
*Chairman Legislative Committee, N. D. A.*

